# MRC ORACLE Children Study. Long term outcomes following prescription of antibiotics to pregnant women with either spontaneous preterm labour or preterm rupture of the membranes

**DOI:** 10.1186/1471-2393-8-14

**Published:** 2008-04-24

**Authors:** Sara Kenyon, Peter Brocklehurst, David Jones, Neil Marlow, Alison Salt, David Taylor

**Affiliations:** 1 Reproductive Sciences Section, University of Leicester, Leicester, LE2 7LX, UK; 2 NPEU, Institute of Health Sciences, Old Road, Headington, Oxford, OX3 7LF, UK; 3 Department of Health Sciences, University of Leicester, LE2 7LX, UK; 4 Child Health, Queens Medical Centre, Nottingham, NG7 2UH, UK; 5 Great Ormond Street Hospital for Sick Children, Great Ormond Street, London, WC1N 3JH, UK

## Abstract

**Background:**

The Medical Research Council (MRC) ORACLE trial evaluated the use of co-amoxiclav 375 mg and/or erythromycin 250 mg in women presenting with preterm rupture of membranes (PROM) ORACLE I or in spontaneous preterm labour (SPL) ORACLE II using a factorial design. The results showed that for women with a singleton baby with PROM the prescription of erythromycin is associated with improvements in short term neonatal outcomes, although co-amoxiclav is associated with prolongation of pregnancy, a significantly higher rate of neonatal necrotising enterocolitis was found in these babies. Prescription of erythromycin is now established practice for women with PROM. For women with SPL antibiotics demonstrated no improvements in short term neonatal outcomes and are not recommended treatment. There is evidence that both these conditions are associated with subclinical infection so perinatal antibiotic administration may reduce the risk of later disabilities, including cerebral palsy, although the risk may be increased through exposure to inflammatory cytokines, so assessment of longer term functional and educational outcomes is appropriate.

**Methods:**

The MRC ORACLE Children's Study will follow up UK children at age 7 years born to 4809 women with PROM and the 4266 women with SPL enrolled in the earlier ORACLE trials. We will use a parental questionnaire including validated tools to assess disability and behaviour. We will collect the frequency of specific medical conditions: cerebral palsy, epilepsy, respiratory illness including asthma, diabetes, admission to hospital in last year and other diseases, as reported by parents.

National standard test results will be collected to assess educational attainment at Key Stage 1 for children in England.

**Discussion:**

This study is designed to investigate whether or not peripartum antibiotics improve health and disability for children at 7 years of age.

**Trial registration:**

The ORACLE Trial and Children Study is registered in the Current Controlled Trials registry. ISCRTN 52995660

## Background & Rationale

The sequaelae of preterm birth pose a significant public health problem. Children born before 37 weeks gestational age are at increasing risk of major disabilities, such as cerebral palsy, with decreasing gestation [1]. Furthermore, of the preterm children without disability, many will have important behavioural and educational difficulties. The ORACLE Children Study presents a unique opportunity to determine if a simple, inexpensive intervention reduces the long term sequaelae of preterm birth with benefit for individuals and their families.

The ORACLE trial evaluated the prescription of Co-amoxiclav 375 mg and/or erythromycin 250 mg to women either with PROM [2] or in SPL [3]. The trial reported that erythromycin did improve the composite primary outcome (death or major cerebral abnormality on ultrasound prior to discharge or chronic lung disease defined as needing oxygen therapy at 36/40 post conceptual age) in singleton babies born to women after PROM. The prescription of Erythromycin was also associated with improvement in other significant outcomes such as prolongation of pregnancy, reduction in the need for neonatal ventilation, oxygenation and treatment with surfactant, a decrease in oxygen dependence at 28 days of age or older, and fewer positive neonatal blood cultures. Although Co-amoxiclav was associated with prolongation of pregnancy, a significantly higher incidence of neonatal necrotising enterocolitis was found in these babies. In the group of women with SPL the use of neither Co-amoxiclav nor erythromycin were associated with any improvement in neonatal morbidity or mortality.

Following the ORACLE trial antibiotic treatment for preterm, prelabour rupture of the membranes (PROM) may become standard practice and so there will never be another opportunity to undertake this study and provide unbiased and reliable evidence on this subject.

There is increasing evidence that, in addition to preterm birth, perinatal infection is an independent antecedent of other disability, particularly cerebral palsy and chronic lung disease. Therefore, perinatal administration of antibiotics could prevent neurological and respiratory disability by two mechanisms, either by prolonging pregnancy and/or by eliminating infection. In contrast however, it is possible that prolongation of pregnancy may increase rather than decrease the risk of disability by continuing fetal exposure to inflammatory cytokines, which have also been implicated in the genesis of neurological damage [4] and chronic lung disease [5]. Some clinical evidence to support this possibility comes from two observational studies of neurodevelopmental outcome in preterm infants born after PROM which showed a higher risk of cerebral palsy or neurological impairment compared with gestation matched controls [6,7]. In addition, duration of membrane rupture had a direct relationship with the risk of neurological impairment [7].

The cerebral and respiratory benefits reported with erythromycin in women with PROM in the ORACLE trial may have been secondary to (a) prolongation of pregnancy and (b) a reduction of the effects of fetal and neonatal lung infection or inflammation. Supportive evidence for the latter comes from broncho-alveolar lavage fluid studies. Studies of the constituents of broncho alveolar lavage fluid have shown that infants who develop chronic lung disease have higher concentrations of neutrophils [8], proinflammatory cytokines[9,10] and proxy markers of neutrophil recruitment, than those who recover from respiratory distress syndrome[9,11]. There is also evidence that intrauterine lung inflammation is implicated in the genesis of chronic lung disease, since high concentrations of the potent profibrotic agent, transforming growth factor, have been described in the first broncho-alveolar lavage fluid after birth of infants who go on to develop chronic lung disease [5,12]. The MRC ORACLE Children Study provides a unique opportunity to determine whether the reductions in chronic lung disease found at discharge from hospital in the erythromycin group are reflected in a decreased incidence of respiratory disease and the need for therapy at 7 years of age.

Follow-up of the infants of women who presented with SPL is important, even in the absence of an apparent effect of antibiotics from ORACLE II [3] on short term morbidity. Many clinicians and researchers are convinced from observational evidence that a substantial proportion of SPL is caused by sub-clinical infection [13]. If this evidence is indeed correct then differences in childhood neurodevelopment and respiratory function could be detected between children born to women randomised to antibiotics compared with those randomised to placebo even though crude measures of early neurological damage or respiratory disease were no different between the groups during the perinatal period.

## Methods/Design

ORACLE was a double blind randomised controlled factorial design trial which evaluated whether giving broad spectrum antibiotics to women in SPL or with PROM improved neonatal mortality and morbidity.

Women were randomised to receive antibiotics and matching placebos 4 times daily for 10 days or until delivery, whichever was sooner. ORACLE tested Co-amoxiclav 375 mg and erythromycin 250 mg singularly and in combination.

This phase of the study – the MRC ORACLE Children Study – will follow-up children of women randomised to ORACLE at 7 years of age to determine whether antibiotics have effects on their development, educational attainment, and the risk of conditions such as cerebral palsy and respiratory illness. The MRC ORACLE Children Study will include the 8942 children born to those women randomised in the UK.

### Primary & secondary outcomes

The primary outcome is:

1) the overall level of disability (severe, moderate, mild, none) derived from the Multi-Attribute Health Status (MAHS) classification system.

The secondary outcomes are:

1) number of children with 3 or more abnormal attributes derived from the MAHS classification system

2) degree of function (severe, moderate, mild, none) within the 9 domains of vision, hearing, speech, ambulation, dexterity, emotions, cognition, pain and self care.

3) behaviour (Goodman's strengths and difficulties questionnaire).

4) educational achievement in reading, mathematics and writing at Key Stage 1. Data on both levels and raw scores will be collected.

5) incidence of specific medical conditions:

• cerebral palsy

• epilepsy

• respiratory illness including asthma

• diabetes

• admission to hospital in last year

• other diseases

• death – from trial entry to aged 7 years

- from initial discharge from hospital to aged 7 years

• covariates – family demographic information (housing status, ethnic group, smoking practice) will be collected.

### Inclusion/exclusion criteria

#### Inclusions

• surviving children of mothers recruited to the ORACLE trial

• child resident in UK

#### Exclusions

• recruited outside UK

• adopted or fostered children and children who have emigrated

Children who have been adopted or fostered will not be followed-up to avoid potential distress to parents and child. Children born in Scotland, Northern Ireland or Wales have different National Tests to England and therefore will not have data collected to assess educational achievement.

### Postal parent-report questionnaire

A parental questionnaire will be administered to determine the level of disability (severe, moderate, mild, none). This is based on the Multi-Attribute Health Status (MAHS) Mark II and III [14] classification system developed and widely tested by the Departments of Paediatrics and Clinical Epidemiology and Biostatistics of McMaster University, Canada. The additional domain of behaviour is being assessed using the strengths and difficulties questionnaire developed and validated by Robert Goodman [15]. Additional questions will assess the other health related outcomes. Deaths will be categorised hierarchically using the system below developed by PMG and with the advice of Steve Gould, Consultant Pathologist, John Radcliffe Hospital, Oxford

### Death categorisations

1. Congenital abnormality

2. Cancer/Neoplasia

3. Complications of Prematurity

3a. Chronic lung disease

3b. CLD and CNS

3c. Central Nervous System related to prematurity

4. Central Nervous System

5. Infection

6. Miscellaneous

7. Sudden Unexpected Death in Infancy (SUDIs, SIDS and Unascertained)

### National Tests

In England we will employ a unique and innovative approach of using the standard National Tests (Key Stage 1) to assess the children's educational ability supported by the Qualifications and Curriculum Authority (QCA). Dfes will provide anonymised data for test level achieved and ethnicity of all children in each treatment group. For those children for whom parental consent is obtained level data will be obtained from the Local Education Authority (LEA) and the scores for each question will be collected from individual schools. Children in Northern Ireland and Wales have different National Tests and no such tests are conducted in Scotland therefore these children will not have data collected to assess educational achievement.

### Estimated power of study

A total of 8942 babies survived until the end of data collection in England, Scotland, Wales and Northern Ireland. Of these, 4521 were in the SPL group and 4421 were in the PROM group. Not all of these babies will have survived until age 7 years; assuming an 85% response rate to a parental questionnaire, 3843 responses can be expected from the SPL group and 3758 from the PROM group (7601 in total).

Table 1 demonstrates the differences detectable between any two intervention groups for the listed primary measure (e.g. erythromycin vs. erythromycin-placebo) in each of the specified sub-groups (SPL and PROM). The estimates of prevalence of disability are based on data derived using the same instrument (MAHS Mark II) [14] on extremely low birth weight (ELBW) babies compared with a reference group of normal birth weight babies. As the majority of ELBW babies are born less than 28 weeks gestation they will have a higher prevalence of disability than the children included in the MRC ORACLE Children Study. For the measure of '3 or more attributes' affected, an incidence of 28% occurred in the ELBW group compared with 2% in the reference group. As the gestational age of the ORACLE population of children is between these extremes a conservative disability estimate of 5% has been taken as the principal estimate and sensitivity to variation of this value of the power of the study to detect differences is explored in the table. By ranking children into groups by their degree of disability (the number of attributes affected) the power of the study to demonstrate smaller differences will be increased by using tests for trends.

**Table 1:** For each sub-group (PROM or SPL), a response from 3700 babies will allow the following differences to be detected with 80% power:

**Outcome**	Incidence in erythromycin group	Incidence in erythromycin placebo group	Relative risk reduction
**'3 or more attributes affected'**	**3.1%**	**5%**	**38%**
	5.6%	8%	30%
	1.5%	3%	50%

### Analyses plan

The primary analysis will comprise comparison of MAHS disability prevalence by allocated treatment group, through fitting of logistic models including terms indicating allocation to Co-amoxiclav/not and to Erythromycin/not, together with an interaction term. Both HUI2 and HUI3 data will be collected and analysed as they have been designed to complement one another but are based on the same conceptual framework. HUI2 refers to worry and anxiety, HUI3 to happiness versus depression. Similarly while both refer to the degree of severity of pain, HUI2 focuses on the use of analgesia while HUI3 focuses on the disruption of activities. Self-care is only available in HUI2 and dexterity only in HUI3. Sensitivity analyses will include i) logistic modelling of the MAHS level of disability, ii) corresponding models for risk differences, iii) alternative approaches to analysing outcomes from multiple births, and iv) models adjusted for demographic covariates if their balance deteriorates during further follow up, and for other covariates including length of gestation, and year of KS1 assessment and original ORACLE outcomes.

Analyses of secondary outcomes will follow the approach for the primary outcome, appropriately modified where necessary to accommodate continuous or survival measures. Details of the analysis of KS1 data are to be developed in collaboration with colleagues in the agency responsible for collection and interpretation of these data. Explicit allowance for multiplicity of comparisons will be made in interpretation of the results.

### Planned subgroup analyses

Comparisons of primary and secondary outcomes will be undertaken for the infants of women with: (1) SPL – intact membranes; (2) PROM; (3) multiple & singleton pregnancies; (4) >32 week gestation and < 32 week gestation; (5) > < 28 weeks gestation.

### Data collection

The women were aware of the possibility of a Follow-Up Study when they were recruited to ORACLE. We will trace both mother and child through the Office of National Statistics (ONS) and will be informed of the mother or child's death since discharge from hospital to date. We will then send an invitation letter to the address obtained for the woman using the NHS Tracing Service (NSTS). If no response is obtained the child's GP will be contacted to check contact details and ascertain if there is a reason e.g. child is currently in care or non-English speaker etc. Translations of all study materials will be available should they be required and an inexpensive pen will be included with the invitation and information leaflets.

Once we have established contact with the woman and child we will make every effort to maintain this by the use of birthday cards and newsletters (to maintain regular contact) and change of address cards (to facilitate tracking of address changes). We will receive listings from ONS quarterly throughout the study so we are aware of embarkations and deaths are notified immediately. We may consider giving maternity units and GP's surgeries posters and information leaflets so women can contact us directly. We confirm current address using NSTS prior to contact when the child is 7 years old. The Information Leaflet is sent to the parents, and two weeks later a questionnaire. Should no response be obtained this is followed by two letters, a fortnight apart, to the GP to ascertain whether we have the correct address and contact information. Following this confirmation a reminder letter is sent to the parent's/carers the week of the child's 7^th ^birthday including a ~5 voucher which is sent recorded delivery. Should no response be forthcoming the last reminder is done 3 weeks after that and involves either letter or telephone contact, where a phone number is available.

A consent form for educational outcomes (KS1) is sent to parents in England only. KS1 data is to be collected from either schools or Local Education Authority (LEA).

A thank you card will be sent to parents once the questionnaire has been completed and it is planned to send the results of the follow-up to parents, should they request them, in 2008.

### Safety

This study is not actively treating women – it is the follow up of the children. However, clinical care is different for the two groups. It is recommended that women with preterm rupture of the membranes receive erythromycin and women with spontaneous preterm labour are not prescribed antibiotic therapy.

A Data Monitoring Committee (DMC) has been established and will meet annually to consider interim analyses. The Committee will determine if additional interim analyses of trial data should be undertaken. It will consider the data from interim analyses, unblinded if considered appropriate, plus any additional safety issues and relevant information from other sources. In the light of this, and ensuring that ethical considerations are of prime importance, to report (following each DMEC meeting) to the Trial Steering Committee (TSC) and to recommend on the continuation of the study. The DMC will consider any requests for release of interim trial data and to recommend to the TSC on the availability of this and in the event of further funding being required, to provide to the TSC and MRC appropriate information and advice on the data gathered to date that will not jeopardise the integrity of the study.

The DMC membership is Professor Diane Elbourne, Chair, Professor of Health Care Evaluation, London School of Hygiene and Tropical Medicine; Ms Helena McNally, ECMO Trial, NPEU; Professor Martin Whittle, Clinical Co-Director, National Collaborating Centre for Women and Children's Health, London.

### Publication and dissemination of results

The results will be published in scientific journals. A writing committee will consist of all applicants with Sara Kenyon taking the lead on writing the paper. To safeguard the scientific integrity of the study, data should not be presented in public or submitted for publication without the consent from the Trial Steering Committee (see organisation below)

### Organisation and Governance

The protocol and all subsequent amendments received approval from West Midlands Multicentre Research Ethics Committee Date of approval April 2002 Reference number MREC/03/7/14. The study will be sponsored by University Hospitals Leicester. The study will be run according to the MRC Good Clinical Practice Guidelines and UK Data Protection requirements. The study will be monitored on a day-to-day basis by the Study Office based at the University of Leicester with the Project Management Group which consists of all the applicants.

The study will be overseen by a Trial Steering Committee (TSC) with the following terms of reference: to monitor and supervise the progress of the trial MRC ORACLE Children Study towards its interim and overall objectives; to review at regular intervals relevant information from other sources (eg, other related trials); to consider the recommendations of the DMC; in the light of these to inform the Council and relevant Research Boards on the progress of the trial; to advise Council on publicity and the presentation of all aspects of the trial.

#### Membership

Richard Lilford, Chair, Professor of Clinical Epidemiology, University of Birmingham; Dr Catherine Elliott, MRC Head Office; Professor Richard Cooke, Consultant Neonatologist, Liverpool Women's Hospital; Mr Chris Whetton, Assistant Director, National Foundation for Educational Research; Gill Gyte National Childbirth Trust; Dr Phillippa Russell, Director, National Council for Disabled Children; Julie Cahill, School Improvement Advisor, London.

## Discussion

The ORACLE Children Study will provide important information about the health, development and educational attainment of children at 7 years of age whose mothers were exposed to peripartum antibiotics. This information will be important to both parents and clinicians when faced with either preterm ruptured membranes or threatened spontaneous preterm labour.

## Abbreviations

ELBW: Extremely Low Birth Weight; GP: General Practitioner; HUI: Health Utilities Index; KS1: Key Stage 1; LEA: Local Education Authority; MAHS: Multi Attribute Health Status; MRC: Medical Research Council; NSTS: National Strategic Tracing Service; ONS: Office of National Statistics; ORACLE :Overview of the Role of Antibiotics in the Curtailment of Labour and Early delivery; PROM: Preterm Rupture Of the Membranes; QCA: Qualifications and Curriculum Authority; SPL: Spontaneous Preterm Labour.

## Competing interests

The authors declare that they have no competing interests.

## Authors' contributions

All authors contributed to the study design and developed the protocol. SK will led the study and together with DT and PB will contribute knowledge of maternity practice, KP and DJ statistics, NM neonatology and longer term developmental follow-up, and AS Community Paediatrics and long term follow-up.

**Figure 1:**
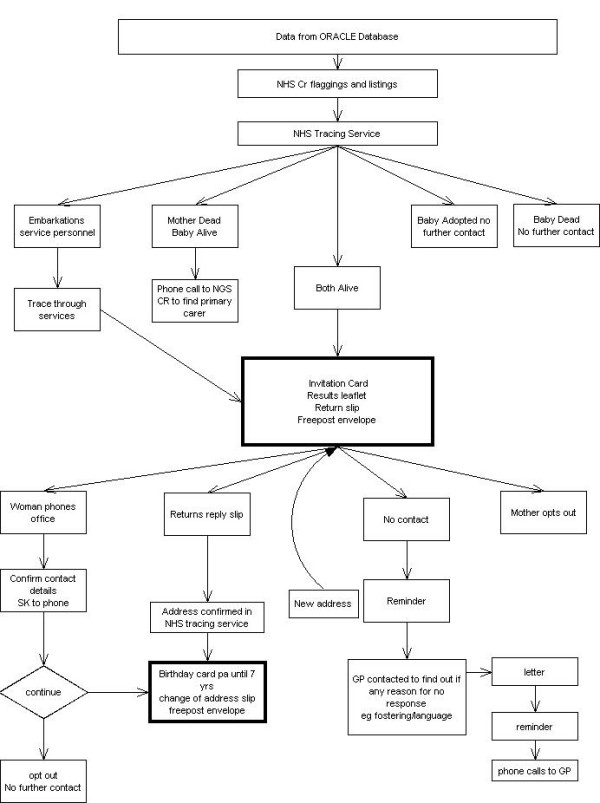
Tracing and contact mechanisms.

**Figure 2:**
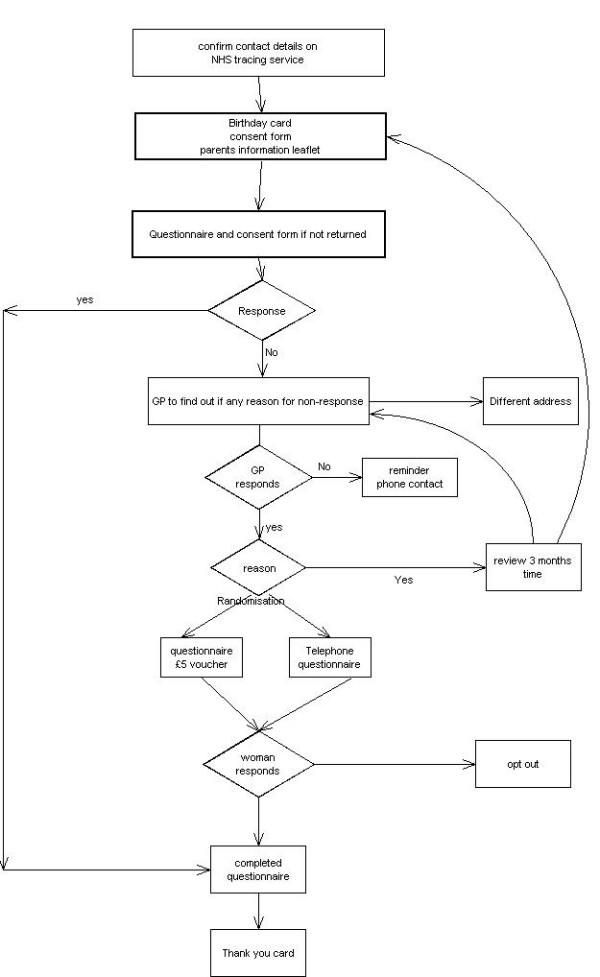
Child in 7^th ^year.

**Figure 3:**
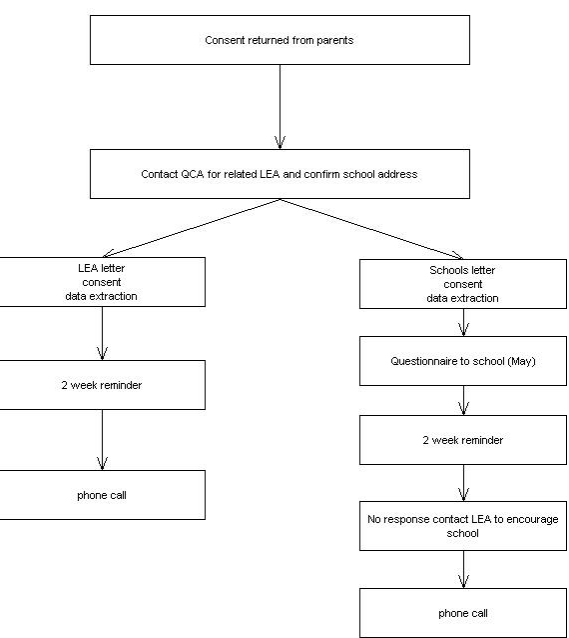
KS1 data extraction.

## Pre-publication history

The pre-publication history for this paper can be accessed here:


